# A Spike Time-Dependent Online Learning Algorithm Derived From Biological Olfaction

**DOI:** 10.3389/fnins.2019.00656

**Published:** 2019-06-27

**Authors:** Ayon Borthakur, Thomas A. Cleland

**Affiliations:** ^1^Computational Physiology Laboratory, Field of Computational Biology, Cornell University, Ithaca, NY, United States; ^2^Computational Physiology Laboratory, Department of Psychology, Cornell University, Ithaca, NY, United States

**Keywords:** SNN, online learning, olfaction, STDP, local learning, spike time coding

## Abstract

We have developed a spiking neural network (SNN) algorithm for signal restoration and identification based on principles extracted from the mammalian olfactory system and broadly applicable to input from arbitrary sensor arrays. For interpretability and development purposes, we here examine the properties of its initial feedforward projection. Like the full algorithm, this feedforward component is fully spike timing-based, and utilizes online learning based on local synaptic rules such as spike timing-dependent plasticity (STDP). Using an intermediate metric to assess the properties of this initial projection, the feedforward network exhibits high classification performance after few-shot learning without catastrophic forgetting, and includes a *none of the above* outcome to reflect classifier confidence. We demonstrate online learning performance using a publicly available machine olfaction dataset with challenges including relatively small training sets, variable stimulus concentrations, and 3 years of sensor drift.

## Introduction

Convolutional networks have enabled tremendous progress in image recognition. However, analogous problems in high-dimensional modalities that lack the two-dimensional internal structure of visual images are not well-addressed by these networks, and the development of brain-mimetic network-based signal identification strategies in such modalities has lagged. This is unfortunate, as there are innumerable applications for such classifiers, including medical screening, genomics, and machine olfaction. Among these, machine olfaction methods have been directly inspired by the mammalian and insect olfactory systems—highly structured and well-studied biological networks that learn rapidly and non-iteratively, utilize local learning rules, resist catastrophic forgetting, can identify and learn new classes of odors (i.e., that do not map to existing representations), and can robustly identify signals of interest in the presence of strong interference. We studied the mammalian olfactory system in order to extract computational principles and algorithms that could underlie its unmatched ability to identify and classify genuinely high-dimensional signals under a variety of challenging conditions.

Most current research effort in machine olfaction is devoted to sensor development, including technologies such as multi-chamber metal oxide semiconductor (MOS) sensors (Gonzalez et al., 2011), high-density polymer sensors (Beccherelli et al., 2010), molecularly imprinted MOS and polymer sensors (Shi et al., 1999; Iskierko et al., 2016; Zhang et al., 2017), and surface acoustic wave sensors (Länge et al., 2008). In an effort to mimic properties of the biological system, there even have been efforts to develop sensors based on G protein-coupled receptor proteins bound to carbon nanotube transistors (Liu et al., 2006). In contrast, there has been relatively little effort spent mining the post-sensory networks of the olfactory system for clues to its unmatched performance, despite a broad understanding that biological odorant receptors are neither particularly specific nor particularly sensitive to odor stimuli. Rather, the power of the biological olfactory system derives from the concerted effects of the large numbers and diversity of its sensors, and by its post-sensory signal processing in the olfactory bulb and related cortices. These core principles inform recent developments in neuromorphic olfaction (Persaud et al., 2013; Schmuker et al., 2015), and have been highlighted in contemporary artificial systems work based on the similarly-structured olfactory system of insects (Schmuker et al., 2014; Mehta et al., 2017; Diamond et al., 2019).

We here present a spiking neural network (SNN)-based online learning algorithm, based on principles and motifs derived from the mammalian olfactory system, that can accurately classify noisy high-dimensional signals into categories that have been dynamically defined by few-shot learning. In order to better interpret the basis for the algorithm's capabilities, the present work focuses entirely on the properties of the first feedforward projection, omitting the spike timing-based feedback loop that forms the core network of the full OB model (Imam and Cleland, 2019). Glomerular-layer processing is represented here by two preprocessing algorithms, whereas plasticity for rapid learning is embedded in subsequent processing by the external plexiform layer (EPL) network. Information in the EPL network is mediated by patterns of spike timing with respect to a common clock corresponding to the biological gamma rhythm, and learning is based on localized spike timing-based synaptic plasticity rules. The algorithm is implemented in PyTorch for GPU computation, but designed for later implementation on state-of-the-art neuromorphic computing hardware (Davies et al., 2018); the initial version of the complete attractor model has been implemented on Intel Loihi (Imam and Cleland, 2019). We here demonstrate the interim performance of the feedforward algorithm using a well-established machine olfaction dataset with distinct challenges including multiple odorant classes, variable stimulus concentrations, physically degraded sensors, and substantial sensor drift over time.

## Core Principles

The network is based on the architecture of the mammalian olfactory bulb (reviewed in Cleland, 2014; Nagayama et al., 2014). Primary olfactory sensory neurons (OSNs) express a single odorant receptor type from a family of hundreds (depending on animal species). The axons of OSNs that express the same receptor type converge to a common location on the surface of the olfactory bulb (OB), forming a mass of neuropil called a glomerulus. Each glomerulus thus is associated with exactly one receptor type, and serves as the basis for an OB *column*. The profile of glomerular activation levels across the hundreds of receptor types (~400 in humans, ~1,200 in rats and mice) that are activated by a given odorant constitutes a high-dimensional vector of sensory input (Zaidi et al., 2013). Within this first (*glomerular*) layer of the OB, a number of preprocessing computations also are performed, including a high-dimensional form of contrast enhancement (Cleland and Sethupathy, 2006) and an intricate set of computations mediating a type of global feedback normalization that enables concentration tolerance (Cleland et al., 2012). The cellular and synaptic properties of this layer also begin the process of transforming stationary input vectors into spike timing-based representations discretized by 30–80 Hz gamma oscillations (Kashiwadani et al., 1999; Li and Cleland, 2017). The EPL, which constitutes the deeper computational layer of the OB, comprises a matrix of reciprocal interactions between principal neurons activated by sensory input (mitral cells; MCs) and inhibitory interneurons (granule cells; GCs). Computations in this layer depend on fine-timescale spike timing (Lepousez and Lledo, 2013) and odor learning (Lepousez et al., 2014; Mandairon et al., 2018), and modify the information exported from the OB to its follower cortices.

**Figure 1:**
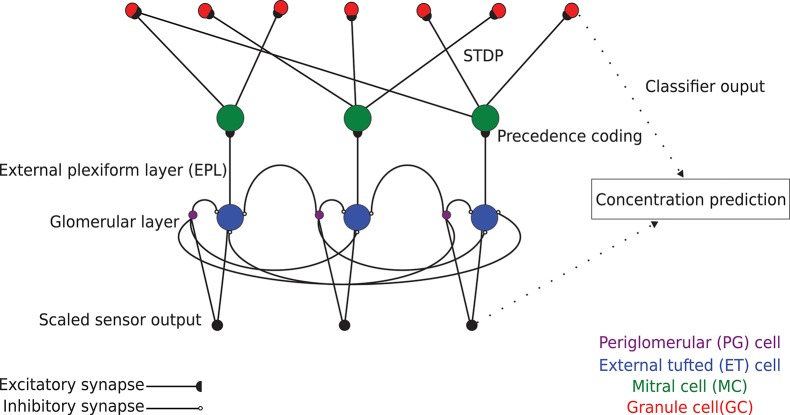
Schematic model of *EPLff* network circuitry (three columns depicted). Sensor-scaled input data are presented in parallel to excitatory external tufted (ET) cells and inhibitory periglomerular (PG) cells in the glomerular layer. This glomerular-layer circuit performs an unsupervised concentration tolerance preprocessor step based on the graded inhibition of ET cells by PG cells. The concentration-normalized ET cell activity then is presented as input to their co-columnar mitral cells (MCs). In the external plexiform layer (EPL), comprising MC interactions with inhibitory granule cells (GCs), levels of sensory input are encoded in MCs as a spike time precedence code across the MC population. MCs project randomly onto GCs with a connection probability of 0.4. These synaptic connections are plastic, following a standard STDP rule that enables GCs to learn high-order receptive fields (Linster and Cleland, 2010). The GC population consequently learns to recognize specific odorants by measuring the similarity of high dimensional GC activity vectors with the Hamming distance metric.

Chemical sensing in machine olfaction is similarly based upon combinatorial coding (Persaud and Dodd, 1982); specificity is achieved by combining the responses of many poorly-selective sensors. In the present algorithm, networks were defined with a number of columns such that each column received input from one type of sensor in the connected input array. Columns each comprised one external tufted (ET) cell and one periglomerular (PG) cell to mediate glomerular-layer preprocessing, and one MC and a variable number of GCs to mediate EPL odorant learning and classification (Figure 1; see section Online Learning). Sensory input was preprocessed by the ET and PG cells of the glomerular layer (for concentration tolerance), and then delivered as excitation to the array of MCs, which generated action potentials. Each MC synaptically excited a number of randomly determined GCs drawn from across the entire network, whereas activated GCs synaptically inhibited the MC in their home column. Importantly, for present purposes, these inhibitory feedback weights were all reduced to zero to disable the feedback loop and EPL attractor dynamics, enabling study of the initial feedforward transformation based on excitatory synaptic plasticity alone. During learning, the excitatory synapses followed a STDP rule that systematically altered their weights, thereby modifying the complex receptive fields of recipient GCs in the service of odor learning. In the present study, in lieu of the modified spike timing of the MC ensemble that characterizes the output of the full model (Imam and Cleland, 2019), the binary vector describing GC ensemble activity in response to odor stimulation (0: non-spiking GC; 1: spiking GC) served as the processed data for classification. Because we here report the capacities of the initial feedforward projection of preprocessed data onto the GC interneuron array within the EPL—an initial transformation that sets the stage for ongoing dynamics not discussed herein—we refer to our present method as the *EPLff* network algorithm.

## Materials and Methods

### Data Preprocessing

#### Sensor Scaling

We defined a set of preprocessing algorithms, any or all of which could be applied to a given data set to prepare it for efficient analysis by the core algorithm. The first of these, *sensor scaling*, is applied to compensate for heterogeneity in the scales of different sensors—for example, an array comprising a combination of 1.8*V* and 5*V* sensors. One simple solution is to scale the responses of each sensor by the maximum response of that sensor. Let *x*_1_, *x*_2_, *x*_3_, …, *x*_*n*_ be the responses of *n* sensors to a given odor and *s*_1_, *s*_2_, *s*_3_, …, *s*_*n*_ be the maximum response values of those sensors. Then, $\frac{x_{1}}{s_{1}},\frac{x_{2}}{s_{2}},\frac{x_{3}}{s_{3}},\ldots ,\frac{x_{n}}{s_{n}}$ represent the sensor-scaled responses. The maximum sensor response vector *S* could be predetermined (as in sensor voltages), or estimated using a model validation set. Here, we defined *S* using the model validation set (10% of Batch 1 data; see section Dataset) and utilized the same value of *S* for scaling all subsequent learning and inference data (see section Sensor Drift). This preprocessing algorithm becomes particularly useful when analyzing data from arbitrary or uncharacterized sensors, or from arrays of sensors that have degraded and drifted non-uniformly over time.

#### Unsupervised Concentration Tolerance

Concentration tolerance is a critical feature of mammalian as well as insect olfaction (Cleland and Sethupathy, 2006; Cleland et al., 2012; Serrano et al., 2013). Changes in odorant concentration evoke non-linear effects in receptor activation patterns that are substantial in magnitude and often indistinguishable from those based on changes in odor quality. Distinguishing concentration differences from genuine quality differences appears to rely upon multiple coordinated mechanisms within olfactory bulb circuitry (Cleland et al., 2012), but the most important of these is a global inhibitory feedback mechanism instantiated in the deep glomerular layer (Cleland et al., 2007; Banerjee et al., 2015). The consequence of this circuit is that MC spike rates are not strongly or uniformly affected by concentration changes, and the overall activation of the olfactory bulb network remains relatively stable. We implemented this concentration tolerance mechanism as the graded inhibition of external tufted cells (ET) by periglomerular cell (PG) interneurons in the OB glomerular layer (Figure 1)—a mechanism based upon recent experimental findings in which ET cells serve as the primary gates of MC activation (Gire et al., 2012; Banerjee et al., 2015)—and tested its importance empirically on machine olfaction data sets. This concentration tolerance mechanism facilitates recognition of odor stimuli even when they are encountered at concentrations on which the network has not been trained; moreover, once an odor has been identified, its concentration can be estimated based on the level of feedback that the network delivers in response to its presentation. This preprocessing step requires no information about input data labels, and greatly facilitates few-shot learning.

Input from each sensor was delivered directly to PG and ET interneurons associated with the column corresponding to that sensor, and the resulting PG cell activity was delivered via graded synaptic inhibition onto all ET cells within all columns in the network. ET cells in turn then synaptically excited their corresponding, cocolumnar MCs (Figure 1). The approximate outcome of this preprocessor algorithm is as follows: given that $x_{1}^{ET},x_{2}^{ET},x_{3}^{ET},\ldots ,x_{n}^{ET}$ denote the responses of ET cells to odor inputs (prior to their inhibition by PG cells), and $x_{1}^{pg},x_{2}^{pg},x_{3}^{pg},\ldots ,x_{n}^{pg}$ denote the analogous responses of PG interneurons to these same inputs, the resulting input to MC somata from ET cells following their PG-mediated lateral inhibition will be

(1) $$\begin{array}{l} \frac{x_{1}^{ET}}{\sum x^{pg}},\frac{x_{2}^{ET}}{\sum x^{pg}},\frac{x_{3}^{ET}}{\sum x^{pg}},\ldots ,\frac{x_{n}^{ET}}{\sum x^{pg}} \end{array}$$

A version of this algorithm has been implemented using spiking networks on IBM TrueNorth neuromorphic hardware (Imam et al., 2012).

### Core Algorithm

#### Cellular and Synaptic Models

We modeled the MCs and GCs as leaky integrate-and-fire neurons with an update period of 0.01 ms. The evolution of the membrane potential *v* of MCs and GCs over time was described as

(2) $$\begin{array}{l} \tau \frac{dv}{dt}=-v+IR \end{array}$$

where τ = *r*_*m*_*c*_*m*_ was the membrane time constant and *r*_*m*_ and *c*_*m*_ denote the membrane resistance and capacitance, respectively. For MCs, the input current *I* corresponded to sensory input received from ET cells (after preprocessing by the ET and PG neurons of the glomerular layer; Figure 1), whereas for GCs, *I* constituted the total synaptic input from convergent presynaptic MCs. In GCs, the parameter *R* was set to equal *r*_*m*_, whereas in MCs it was set to *r*_*m*_/*r*_*shunt*_, where *r*_*shunt*_ was the oscillatory shunting inhibition of the gamma clock (described below). When *v* ≥ *v*_*th*_, where *v*_*th*_ denotes the spike threshold, a spike event was generated and *v* was reset to 0. The total excitatory current to GCs was modeled as

(3) $$\begin{array}{l} I=g_{w}\left(E_{n}-v\right) \end{array}$$

where *E*_*n*_ was the Nernst potential of the excitatory current (+70*mv*), *v* was the GC membrane potential, and $g_{w}=\overset{n}{\underset{i=1}{\sum }}w_{i}g_{\max}\frac{\tau_{1}\tau_{2}}{\tau_{1}-\tau_{2}}\left(e^{\frac{-\left(t-t_{i}\right)}{\tau_{1}}}-e^{\frac{-\left(t-t_{i}\right)}{\tau_{2}}}\right)$ describes the open probability of the AMPA-like synaptic conductances. Here, *t*_*i*_ denotes presynaptic spike timing, *w*_*i*_ denotes the synaptic weight, and *g*_max_ is a scaling factor.

The parameters *c*_*m*_, *r*_*m*_, *r*_*shunt*_, *E*_*n*_, *g*_max_, τ_1_, and τ_2_ were determined only once each for MCs and GCs using a synthetic data set (Borthakur and Cleland, 2017) and remained unchanged during the application of the algorithm to real datasets. The value of *w*_*i*_at each synapse also was set to a fixed starting value based on synthetic data, but was dynamically updated according to the STDP learning rule. The spiking thresholds *v*_*th*_ of MCs and GCs were determined by assessing algorithm performance on the training and validation sets. Because we observed that using heterogeneous values of *v*_*th*_ across GCs improved performance, the values of *v*_*th*_ were randomly assigned across GCs from a uniform distribution.

#### Gamma Clock and Spike Precedence Code

Oscillations in the local field potential are observed throughout the brain, arising from the synchronization of activity in neuronal ensembles. In the OB, gamma-band (30–80 Hz) oscillations are associated with the coordinated periodic inhibition of MCs by GCs (Li and Cleland, 2017; Peace et al., 2017) that constrains MC spike timing (Kashiwadani et al., 1999), thereby serving as a common clock. For this work, we modeled a single cycle gamma oscillation as a sinusoidal shunting inhibition *r*_*shunt*_ delivered onto all MCs,

(4) $$\begin{array}{l} r_{shunt}=-3.8^{*}\cos\left(\frac{2\pi^{*}f^{*}t}{1000}\right)+5 \end{array}$$

where *f* is the oscillation frequency (40 Hz) and *t* is the simulation time. We used a spike precedence coding scheme for MCs (Panzeri et al., 2010) where earlier MC spike phases correspond to stronger sensor input and are correspondingly more effective at growing and maintaining spike timing-dependent plastic synapses (Linster and Cleland, 2010). In the full model, the gamma clock serves as the iterative basis for the attractor; for present purposes in the *EPLff* context it served only to structure the spike times of active MCs converging onto particular GCs (precedence coding), and thereby to govern the changes in excitatory synaptic weights according to the STDP rule (see below).

#### Connection Topology

MC lateral dendrites support action potential propagation to GCs across the entire extent of the OB (Xiong and Chen, 2002; Peace et al., 2017), whereas inhibition of MCs by GCs is more localized. Excitatory MC-GC synapses were initialized with a uniformly distributed random probability *cp* of connection and a uniform weight *w*_0_; synaptic weights were modified thereafter by learning. The initial connection probability *cp* was determined using a synthetic data set (Borthakur and Cleland, 2017), and was set to *cp* = 0.4 in the present simulations. For present purposes, as noted above, GC-MC inhibitory weights were set to zero to disable attractor dynamics.

#### Spike Timing-Dependent Plasticity Rule

We used a modified spike timing-dependent plasticity rule (STDP; Song et al., 2000; Dan and Poo, 2004) to regulate MC-GC excitatory synaptic weight modification. Briefly, synaptic weight changes were initiated by GC spikes and depended exponentially upon the spike timing difference between the postsynaptic GC spike and the presynaptic MC spike. When a presynaptic MC spike preceded its postsynaptic GC spike within the same gamma cycle, *w* for that synapse was increased; in contrast, when MC spikes followed GC spikes, or when a GC spike occurred without a presynaptic MC spike, *w* was decremented. Synaptic weights were limited by a maximum weight *w*_max_. The pairing of STDP with MC spike precedence coding discretized by the gamma clock generated a *k* *winners take all* rule, in which the value of *k* depended substantially on the GC spike threshold *v*_*th*_ and the maximum excitatory synaptic weight *w*_max_. Under this rule, activated GCs were transformed from non-specialized cells receiving weak inputs from a broad and random distribution of MCs into specialized, fully differentiated neurons that responded only to coordinated activation across a specific ensemble of *k* MCs. Under all training conditions, for present purposes, we set a high learning rate such that, after one cycle of learning, each of the synapses could have one of only three values: *w*_0_, *w*_max_, or 0.

The STDP parameters were similar to our previous work using a synthetic data set (Borthakur and Cleland, 2017); among these, only the maximum synaptic weight *w*_max_ was tuned based on validation set performance. For this feedforward implementation, online learning without the requirement of storing training data yielded its best validation set performance when *w*_max_ = *w*_0_, such that learning was limited to long-term synaptic depression (Borthakur and Cleland, 2017).

#### Classification

For the classification of test odorants in this reduced feedforward *EPLff* implementation, we calculated the Hamming distance between the binary vectors of GC odorant representations. Specifically, for every input, GCs generated a binary vector based upon whether the GC spiked (1) or did not spike (0). We matched the similarity of test set binary vectors with the training set vector(s) using the Hamming distance and classified the test sample based upon the label of the closest training sample. Alternatively, an overlap metric between GC activation patterns also was calculated (Equation 6 from Linster and Cleland, 2010); results based on this method were reliably identical to those of the Hamming distance and hence were omitted from this report. Classification was set to *none of the above* if the Hamming distance of the GC binary vectors was >0.5, or if the overlap metric was <0.5.

### Dataset

We tested our algorithm on the publicly available UCSD gas sensor drift dataset (Vergara et al., 2012; Rodriguez-Lujan et al., 2014), slightly reorganized to better demonstrate online learning. The original dataset contains 13,910 measurements from an array of 16 polymer chemosensors exposed to six gas-phase odorants spanning a wide range of concentrations (10–1,000 *ppmv*) and distributed across 10 batches that were sampled over a period of 3 years to emphasize the challenge of sensor drift over time (Table 1). Owing to drift, the sensors' output statistics change drastically over the course of the 10 batches; between this property, the six different gas types, and the wide range of concentrations delivered, this dataset is well-suited to test the capabilities of the present algorithm without exceeding the learning capacity of its feedforward architecture (Figure 1). For the online learning scenario, we sorted each batch of data according to the odorant trained, but did not organize the data according to concentration. Hence, each training set comprised 1–10 odorant stimuli of the same type but at randomly selected concentrations. Test sets always included all six different odorants, again at randomly selected concentrations. For sensor scaling and the fine-tuning of the algorithm, we used 10% of the Batch 1 data as a validation set. The six odorants in the dataset are, in the order of training used herein: ammonia, acetaldehyde, acetone, ethylene, ethanol, and toluene. Batches 3–5 included only five different odorant stimuli, omitting toluene.

**Table 1:** Properties of the UCSD gas sensor drift dataset.

	**Batch 1**	**Batch 2**	**Batch 3**	**Batch 4**	**Batch 5**	**Batch 6**	**Batch 7**	**Batch 8**	**Batch 9**	**Batch 10**
Months	1–2	3–10	11–13	14–15	16	17–20	21	22–23	24–30	36
#Samples	445	1,244	1,586	161	197	2,300	3,613	294	470	3,600

*Months denotes the age of the sensor array during the sampling of the corresponding dataset. #Samples denotes the number of samples provided by the dataset in that particular batch*.

Eight features per chemosensor were recorded in the UCSD dataset, yielding a 128-dimensional feature vector. However, in contrast to previous efforts (Liu et al., 2015; Zhang and Zhang, 2015; Yan et al., 2017; Ma et al., 2018), we chose to use only one feature per sensor in our analysis (the steady state response level), for a total of 16 features. We imposed this restriction to challenge our algorithm, and because generating features from raw data requires additional processing, energy and time, all of which can impair the effectiveness of field-deployable hardware (Yin et al., 2018). Importantly, however, the sensor scaling and concentration tolerance preprocessors described above (section Data Preprocessing) would enable the *EPLff* network to utilize the full 128-dimensional dataset without specific adaptations other than expanding the number of columns accordingly.

## Results

### Data Preprocessing

**Figure 2:**
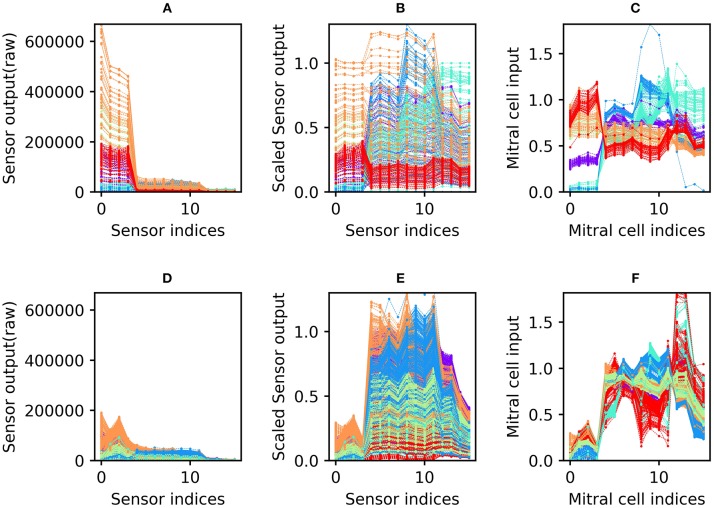
Sensor drift and the application of the sensor scaling and concentration tolerance preprocessors. **(A)** Raw sensory data from the Batch 1 training set. The abscissa denotes the 16 different sensors of the array; the ordinate denotes the magnitude of their responses to specific odorants. The six different colors denote the six odorants of the dataset battery (ammonia, *purple*; acetaldehyde, *blue*; acetone, *aqua*; ethylene, *green*; ethanol, *orange*; and toluene, *red*). Note that each odorant is presented at many different concentrations (Vergara et al., 2012). **(B)** Sensory input from the Batch 1 data shown in **(A)** after preprocessing for sensor scaling. The absolute range of output values is now rendered consistent across all of the sensors in the array. **(C)** Sensory input from the same Batch 1 data after subsequent preprocessing for concentration tolerance by glomerular layer circuitry (Figure 1, ET and PG). The sensory signatures of each of the six odors are now more internally consistent, with less variance owing to the concentration differences inherent in the original data **(D–F)**. As **(A–C)** but with Batch 7 training data. These data were taken from the same set of sensors as depicted in **(A–C)**, but after 21 months of operational degradation, including intermittent periods of use and disuse (Table 1).

All sensory input data were preprocessed before being presented to the network. First, sensor scaling was applied to weight the 16 sensors equally in subsequent computations. The mean raw responses of the 16 sensors differed widely, with some sensors exhibiting an order of magnitude greater variance than others across the 10 odorants tested (Figure 2A). Sensor scaling (Figure 2B) mitigated this effect by scaling each sensor's gain such that the dynamic ranges of all sensors across the test battery were effectively equal. This process enabled each sensor to contribute a comparable amount of information to subsequent computations (up to a limit imposed by each sensor's signal to noise ratio), and improved network performance by maintaining consistent mean activity levels across test odorants.

Since each odorant was presented at a wide range of randomly selected concentrations, the response of the sensor array to a given odorant varied widely across presentations (most clearly observable in Figure 2B). Application of the unsupervised concentration tolerance preprocessor sharply and selectively reduced the concentration-specific variance among responses to presented odorants (Figure 2C). These preprocessed odorant signatures then were presented to the plastic *EPLff* network for training or classification. Notably, this preprocessor step greatly facilitated cross-concentration odorant recognition, even enabling the accurate classification of samples presented at concentrations that were not included in the training set. This was particularly important for one- and few-shot learning, in which the network was trained on just one or a few exemplars (respectively), at unknown concentration(s), such that most of the odorants in the test set were presented at concentrations on which the network had never been trained.

The sensor scaling preprocessor (retaining the scaling factors determined from the 10% validation set of Batch 1), combined with the normalization effects of the subsequent concentration tolerance preprocessor, had the additional benefit of restoring the dynamic range of degraded sensors in order to better match classifier network parameters. Because of this, the network did not need to be reparameterized to effectively analyze the responses of the degraded sensors in the later batches of this dataset. Compared to the raw sensor output of Batch 1 (Figure 2A; collected from new sensors), the raw sensor output of Batch 7 (Figure 2D; collected after 21 months of sensor deterioration) was reduced to roughly a third of its original range. Sensor scaling (Figure 2E) mitigated this effect by magnifying sensor responses into the dynamic range expected by the network. Subsequent preprocessing for concentration tolerance effectively reduced concentration-specific variance, revealing a set of odorant profiles (Figure 2F) that, while qualitatively dissimilar to their profiles based on the same sensors 21 months prior (Figure 2C), appear only modestly degraded in terms of their distinctiveness from one another.

**Figure 3:**
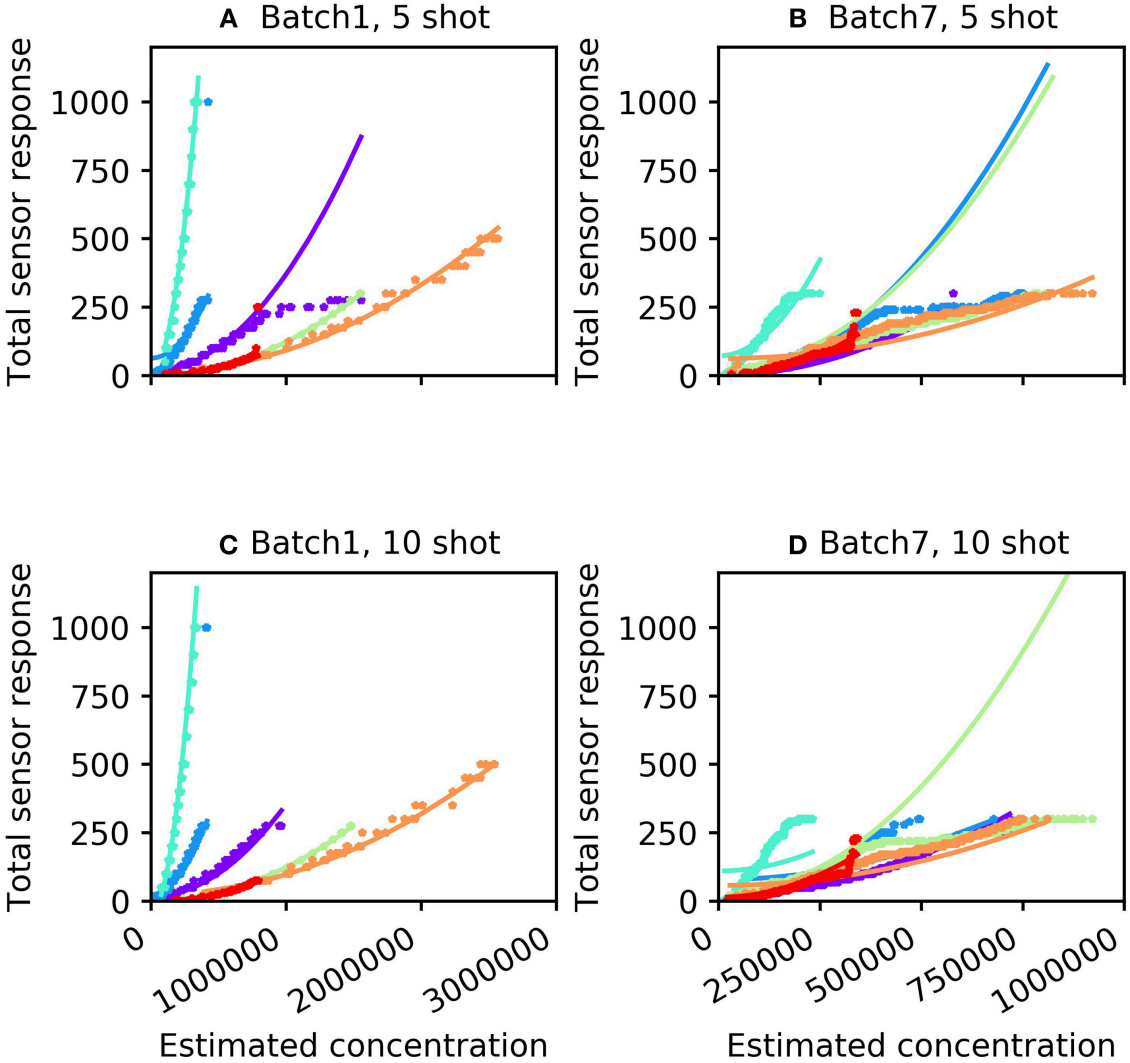
Concentration response function predicted by the algorithm (curves) compared with measured sensor responses across multiple concentrations (stars). **(A)** Batch 1 data with five-shot training. **(B)** Batch 7 data with five-shot training. **(C)** Batch 1 data with 10-shot training. **(D)** Batch 7 data with 10-shot training. The colors denoting particular odorants are the same as in Figure 2.

For many machine olfaction applications, it is useful to estimate the concentrations of gases in the vicinity of the sensors. We sought to use the information extracted from the concentration tolerance preprocessor to estimate the concentrations of test samples after classification. The concentration estimation curve was a function of both odorant identity and the total sensor response profile. Using the sum of the 16 sensor responses (*S*), we fitted an odorant-specific quadratic curve for an implicit model of response profiles across concentrations *C*:*C* = *ax*^2^ + *b*, where the parameters *a* and *b* were determined from the training set. Figure 3 illustrates total sensor responses across concentrations compared to this theoretical prediction for all six odorant gases in Batches 1 and 7. The mean absolute error (MAE) of the prediction (in *ppmv*) was estimated as

(5) $$\begin{array}{l} \frac{\underset{n}{\sum }|C_{pred}-C_{actual}|}{n} \end{array}$$

where *n* denotes the total number of samples. For the five-shot training of Batch 1 (i.e., five random samples drawn from Batch 1 for each odorant), the MAE was 35.14 units (Table 2). This error was reduced to 23.35 for 10-shot learning (Table 2). Similarly, the MAE for Batch 7 decreased from 76.60 (five-shot) to 58.18 (10-shot). To the best of our knowledge, this is the first parallel network architecture to provide an estimate of concentration along with concentration tolerance.

**Table 2:** Concentration estimation performance on test sets of all batches of UCSD gas sensor drift dataset for 5- and 10-shot learning (see Figure 3).

	**Batch 1**	**Batch 2**	**Batch 3**	**Batch 4**	**Batch 5**	**Batch 6**	**Batch 7**	**Batch 8**	**Batch 9**	**Batch 10**	**Mean error**
5 shot	35.14	51.39	32.00	44.73	66.02	37.01	76.60	17.89	0.34	71.06	43.22
10 shot	23.35	33.60	28.97	35.75	64.71	25.23	58.18	19.66	0.84	52.61	34.29

*Concentrations were estimated using the predicted labels and raw sensor input. Errors represent experimental deviation from the predicted quadratic concentration curves and are in units of ppmv*.

### Online Learning

Unlike biological odor learning, artificial neural networks optimized for a certain task tend to suffer from catastrophic forgetting, and the pursuit of online learning capabilities in deep networks is a subject of active study (McCloskey and Cohen, 1989; Kemker and Kanan, 2017; Kirkpatrick et al., 2017; Velez and Clune, 2017; Zenke et al., 2017; Serrà et al., 2018). In contrast, the *EPLff* learning network described herein naturally resists catastrophic forgetting, exhibiting powerful online learning using a fast spike timing-based coding metric. Moreover, we include a *none of the above* outcome which permits classification only above a threshold level of confidence (Huerta and Nowotny, 2009). Hence, after being trained on one odorant, the network could identify a test sample as either that odorant or *none of the above*. After subsequently training the network on a second odorant, it could classify a test sample as either the first trained odorant, the second trained odorant, or *none of the above*. This online learning capacity enables *ad hoc* training of the network, with intermittent testing if desired, with no need to train on or even establish the full list of classifiable odorants in advance. It also facilitates training under missing data conditions (e.g., batches 3–5 contain samples from only five odorants, unlike the other batches which include six odorants), and could be utilized to trigger new learning in an unsupervised exploration context. Finally, once learned, the training set data need not be stored.

**Figure 4:**
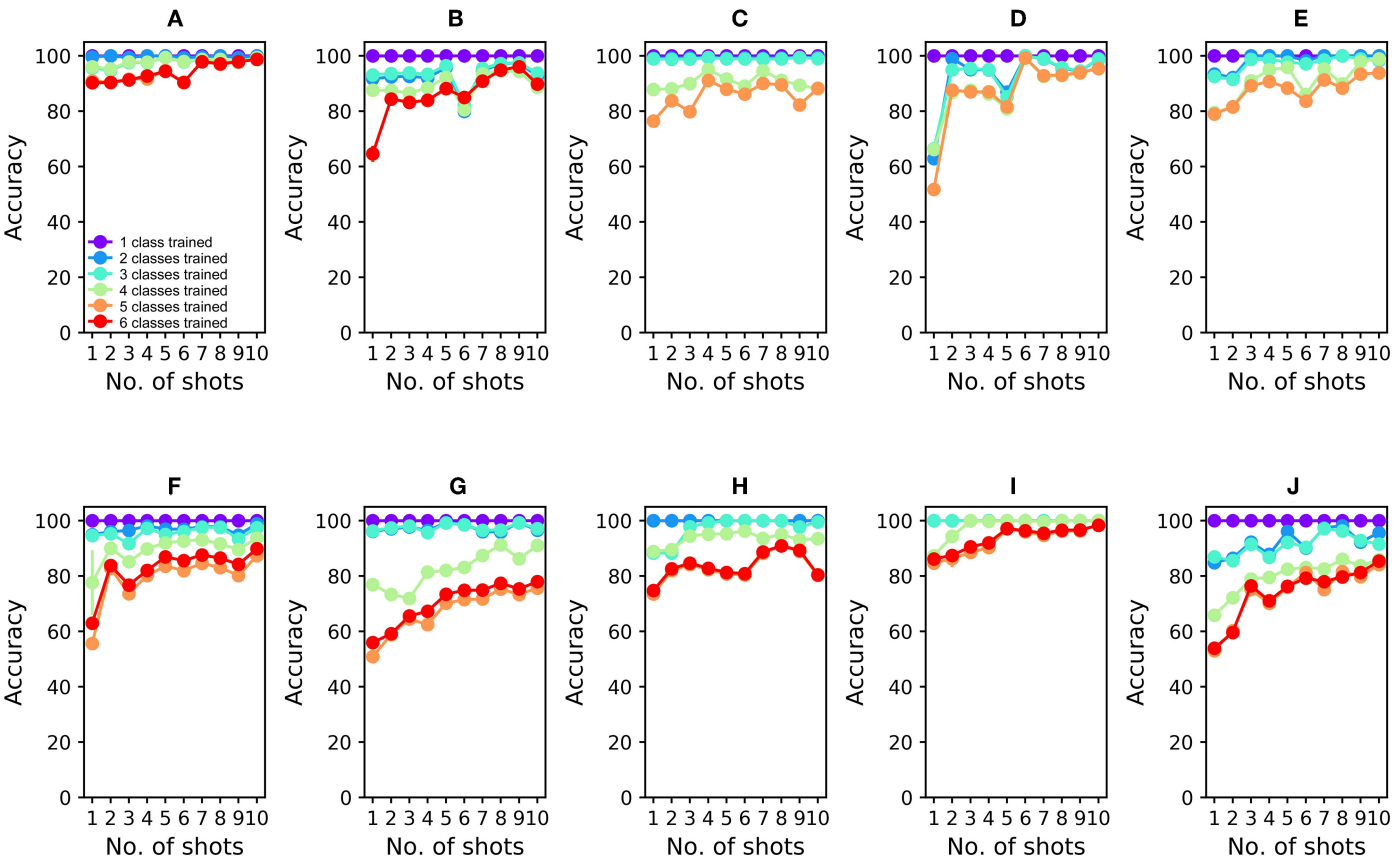
*EPLff* algorithm performance on UCSD gas sensor drift dataset. **(A)** Classification performance during online training and testing of Batch 1 data. Plotted values depict the average classification performance for all test samples of the trained classes. Ammonia was trained first; the purple plot (*One trained class*) denotes the classification accuracy of ammonia test samples as either *ammonia* or *none of the above*. Acetaldehyde was trained second; the blue plot (*Two trained classes*) denotes the classification accuracy of trained-class samples as either *ammonia, acetaldehyde*, or *none of the above*. Online training proceeded with acetone (aqua), ethylene (green), ethanol (orange), and toluene (red) in that order, with the final plot (red or orange) finally denoting the average classification accuracy of all samples into one of the six (or five) odorant classes or, potentially, as *none of the above*. Classification performance degraded slightly as the number of trained odorant representations in the network increased, but improved as the number of learning shots increased **(B)**. As **(A)**, but after training and testing with Batch 2 data **(C–J)**. As **(A,B)**, but after training and testing with Batch 3–10 data, in corresponding order. The colors denoting particular odorants are consistent with Figures 2, 3.

To analyze the 16-sensor UCSD dataset, we constructed a 16-column spiking network with 4800 GC interneurons and a uniformly random MC-GC connection probability *cp* = 0.4. This number of GCs was selected because it was the smallest network that achieved asymptotic performance on the validation dataset (Batch 1, one-shot learning; Table 3). We then trained this network on ammonia using 10 different few-shot training schemes: one-shot, two-shot, three-shot, up through 10-shot in order to measure the utility of additional training. Test data (across all trained odorants and all concentrations in the dataset) were classified with 100.0% accuracy in all cases (Figure 4A; average of three runs). We subsequently trained each of these trained networks on acetaldehyde, using the same number of training trials in each case. After one-shot learning of acetaldehyde, the network classified all trained odorants with 99.61 ± 0.28% accuracy (average of three runs). After subsequent one-shot learning of acetone, classification performance was 95.65 ± 0.19%; after ethylene, 96.06 ± 0.17%; after ethanol, 90.94 ± 0.0%, and finally, after one-shot training on the sixth and final odorant, toluene, test set classification performance across all odorants was 90.27 ± 0.12%. Multiple-shot learning generally produced correspondingly higher classification performance as the training regimen expanded (Figure 4A). Classification using an overlap metric (Linster and Cleland, 2010) rather than the Hamming distance yielded almost identical results (not shown). Critically, classification performance did not catastrophically decline as additional odorants were learned in series (Figure 4, *purple* to *red (orange)* traces in order), particularly when higher-quality sensors were used (Figures 4A–E) or when larger multiple-shot training sets were employed (Figure 4, panel abscissas). These results illustrate that the *EPLff* network, even in the absence of the full model's recurrent component, exhibits true online learning.

**Table 3:** Effect of increased numbers of GCs in the network (GC vector length) on *EPLff* classification accuracy by the Hamming distance criterion, based on one-shot learning using the Batch 1 validation set.

**#GC**	**1 class**	**2 classes**	**3 classes**	**4 classes**	**5 classes**	**6 classes**
	**trained**	**trained**	**trained**	**trained**	**trained**	**trained**
160	100.0	100.0	95.65	96.15	85.71	84.44
1,600	100.0	100.0	95.65	96.15	85.71	86.67
4,800	100.0	100.0	95.65	96.15	85.71	88.89
9,600	100.0	100.0	95.65	96.15	85.71	88.89
14,400	100.0	100.0	95.65	96.15	85.71	88.89
19,200	100.0	100.0	95.65	96.15	85.71	88.89

*Connection probabilities and initial synaptic weights were consistent across all simulations*.

The availability of data in the UCSD dataset from over 3 years of sensor deterioration enabled the testing of this online learning algorithm with both fresh and degraded sensor arrays. Figures 4B–J presents classification results from the same procedures described above but using progressively older and more degraded sensors (Batches 2–10; Table 1; Vergara et al., 2012). Classification performance declined overall as the sensors deteriorated in later batches (Figures 4F–J), but could be substantially rescued by expanding the training regimen from one-shot to few-shot learning. Overall, multiple-shot training reliably improved classification performance, though the residual variance across different training regimes suggests that the random selection of better or poorer class exemplars for training (particularly noting the uncontrolled variable of concentration) exerted a measurable effect on performance (Figure 4; Table 4).

**Table 4:** Mean *EPLff* classification accuracies across all test odorants on the UCSD drift data set by the Hamming distance criterion.

	**Batch 1**	**Batch 2**	**Batch 3**	**Batch 4**	**Batch 5**	**Batch 6**	**Batch 7**	**Batch 8**	**Batch 9**	**Batch 10**	**Average**
1 shot	95.42	83.62	92.48	69.43	88.89	80.97	79.37	87.59	93.04	74.11	84.49
2 shot	95.15	90.38	93.97	93.68	89.35	91.35	80.98	90.41	94.68	77.36	89.73
3 shot	96.28	89.91	93.60	93.02	95.78	87.30	82.98	93.52	96.51	85.72	91.46
4 shot	96.62	90.29	97.02	92.64	96.87	91.25	83.89	93.26	97.02	82.58	92.14
5 shot	97.99	93.47	95.54	86.94	96.40	92.42	87.35	92.89	99.05	87.19	92.92
6 shot	96.00	85.44	94.70	99.69	93.01	92.22	87.77	92.93	98.73	87.36	92.78
7 shot	98.80	94.22	96.54	96.66	97.03	93.47	87.79	95.11	98.30	88.39	94.63
8 shot	98.59	96.45	95.79	95.48	95.64	92.78	89.43	96.16	98.81	90.34	94.95
9 shot	98.39	96.92	94.11	95.35	98.06	90.37	88.92	94.84	98.82	88.32	94.41
10 shot	99.39	92.44	94.95	97.73	98.22	94.55	89.74	92.30	99.48	90.46	94.93

*Odorant-specific classification accuracies are depicted in Figure 4*.

Batch 10 of the UCSD dataset poses a relatively challenging classification problem. To produce it, the sensors were intentionally degraded and contaminated by turning off sensor heating for 5 months following the production of Batch 9 data (Vergara et al., 2012). Prior work with this dataset has achieved up to 73.28% classification performance on Batch 10, without online learning and using a highly introspective approach tailored for this specific dataset (Yan et al., 2017). In contrast, 10-shot learning on Batch 10 using the present *EPLff* algorithm achieved 85.43% classification accuracy.

**Figure 5:**
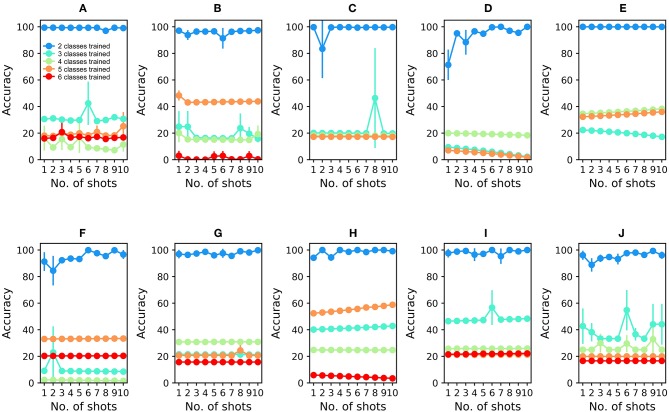
Multilayer perceptron (MLP) performance on UCSD gas sensor drift dataset during online learning. **(A)** Classification performance during online training and testing of Batch 1 data. The network was first trained with ammonia and acetaldehyde (see text); the blue plot denotes the classification accuracy of test samples of these two odorants. Online training proceeded with acetone (aqua), ethylene (green), ethanol (orange), and toluene (red) in that order, with the final plot denoting the average classification accuracy of all samples into one of the five (or four) odorant classes. Unlike the *EPLff* algorithm, the MLP suffered catastrophic forgetting after training on new sample types. **(B–J)** MLP performance during online training and testing of Batch 2–10 data, in corresponding order. Except for the combination of ammonia and acetaldehyde in the first training set, the colors denoting particular odorants are consistent with Figures 2–4.

To compare the *EPLff* network's resistance to catastrophic forgetting against an existing standard method, we built a 16-input multi-layer perceptron (MLP) comprising 16 input units for raw sensor input (ReLu activation), 4,800 hidden units (ReLu activation), and six output units for odorant classification. The MLP was trained using the Adam optimizer (Kingma and Ba, 2014) with a constant learning rate of 0.001. Since there was no straightforward way of implementing *none of the above* in an MLP, the MLP was only trained using two or more odorants (Figure 5). After initial, interspersed training on two odorants from Batch 1, the MLP classified test odorants at high accuracy (99.41 ± 0.0%; average of three runs; Figure 5A). However, its classification accuracy dropped sharply after the subsequent, sequential learning of odorant 3 (30.61 ± 0.0% accuracy), odorant 4 (16.24 ± 9.29%), odorant 5 (18.13 ± 0.0%), and odorant 6 (15.99 ± 0.0%) (Figure 5). Catastrophic forgetting is a well-known limitation of MLPs, and is presented here simply to quantify the contrast in online learning performance between the *EPLff* implementation and a standard network of similar scale.

### Online Reset Learning for Mitigating Sensor Drift

One of the most challenging problems of machine olfaction is *sensor drift*, in which the sensitivity and selectivity profiles of chemosensors gradually change over weeks to months of use or disuse. Efforts to compensate for this drift have taken many forms, from simply replacing sensors to designing highly introspective or specific corrective algorithms. For example, one approach requires the non-random, algorithmically guided selection of relevant samples across batches and/or the utilization of test data as unlabeled data for additional training (Zhang and Zhang, 2015; Yan et al., 2017; Ma et al., 2018). Despite some partial successes in these approaches, the real-world challenge of sensor drift is a fundamentally ill-posed problem, in which the rapidity and nature of functional drift is highly dependent on the idiosyncratic chemistry of individual sensors and specific sensor-analyte pairs.

We argue that the most practical solution to this challenge is to retrain the network as needed to maintain performance, leveraging its rapid, online learning capacity. Specifically, MC-GC synaptic weights are simply reset to their untrained values and the network then is rapidly retrained using the new (degraded) sensor response profiles (*reset learning*). Retraining is not a new approach, of course, but overtly choosing a commitment to heuristic retraining as the primary method for countering sensor drift is important, as it determines additional criteria for real-world device functionality that candidate solutions must address, such as the need for rapid, ideally online retraining in the field and potentially a tolerance for lower-fidelity training sets. Specifically, retraining a traditional classification network may require:

1. Prior knowledge of the number of possible odor classes to be identified,
2. A sufficiently large and representative training set incorporating each of these classes,
3. The retuning of network hyperparameters to match the altered characteristics of the degraded sensors, requiring an indeterminate number of training iterations.

The *EPL* network is not constrained by the above requirements. As demonstrated above, it can be rapidly retrained using small samples of whatever training sets are available and then be updated thereafter—including the subsequent introduction of new classes. The storage of training data for retraining purposes is unnecessary as the network does not suffer from catastrophic forgetting. Finally, the present network does not require hyperparameter retuning. Here, only the MC-GC weights were updated during retraining (using the same STDP rule); sensor scaling factors and all other parameters were ascertained once, using the 10% validation set of Batch 1, and held constant thereafter. Moreover, the *none of the above* classifier confidence feature facilitates awareness of when the network may require retraining; an increase in *none of the above* classifications provides an initial cue that then can be evaluated using known samples.

To assess the efficacy of this approach, we tested the *EPLff* algorithm on the UCSD dataset framed as a sensor drift problem. The procedure for this approach, and consequently the results, are identical to those of section Online Learning above (Figure 4; Table 4). Importantly, the sensor scaling factors and network parameters were tuned only once, using the validation set from Batch 1, on the theory that the concept of rapid reset was incompatible with a strategy of re-optimizing multiple network hyperparameters. Hence, no parameter changes were permitted, other than the MC-GC excitatory synaptic weights that were updated normally during training according to the STDP rule (In order to avoid duplication of figures, this constraint was observed in the simulations of section Online Learning as well). As described above (Figure 4), Batch 1 training samples from all six odorants again were presented to the network in an online learning configuration, and classification performance then was assessed by Batch 1 test data. MC-GC synaptic weights then were reset to the default values (the *reset*), after which Batch 2 training samples were presented to the network in the same manner, followed by testing with Batch 2 test data including all odorants and concentrations. We repeated this process for batches 3–10. We also assessed post-reset classification performance across all batches based on a maximally rapid reset (i.e., one-shot learning) and compared this to performance after expanded training protocols up through 10-shot learning. All classification performance results (averaged across three full repeats each) are depicted in Figure 4 and Table 4. In general, while modest increases in classification accuracy were observed when the training set size was larger, these results demonstrate scalability, showing that the *EPLff* algorithm classifies large sets of test data with reasonable accuracy even based on small training sets and lacking control over the concentrations of presented odorants.

## Discussion

We present a neural network algorithm that achieves superior classification performance in an online learning setting while not being specifically tuned to the statistics of any particular dataset. This property, coupled with its few-shot learning capacity and SNN architecture, renders it particularly appropriate for field-deployable devices based on learning-capable SNN hardware (Davies et al., 2018; Imam and Cleland, 2019), recognizing that the interim use of the Hamming distance for nearest-neighbor classification in the present *EPLff* framework will not be part of such a deployable system. This algorithm is inspired by the architecture of the mammalian olfactory bulb, but is comparably applicable to any high-dimensional dataset that lacks internal low-dimensional structure.

The present *EPLff* incarnation of the network utilizes one or more preprocessor algorithms to prepare data for effective learning and classification by the core network. Among these is an unsupervised concentration tolerance algorithm derived from feedback normalization models of the biological system (Cleland et al., 2007, 2012; Banerjee et al., 2015), a version of which has been previously instantiated in SNN hardware (Imam et al., 2012). Inclusion of this preprocessor enables our algorithm to quickly learn reliable representations based on few-shot learning from odorant samples presented at different and unknown concentrations. Moreover, the network then can generalize across concentrations, correctly classifying unknown test odorants presented at concentrations on which the network was never trained, and even estimating the concentrations of these unknowns.

The subsequent, plastic EPL layer of the network is based on a high-dimensional projection of sensory input data onto a network of interneurons known as granule cells (GCs). In the present feed-forward implementation, our emphasis is on the roles and capacities of two sequential preprocessor steps followed by the STDP-driven plasticity of the excitatory MC-GC synapses. Subsequent extensions of this work will restore the feedback architecture of the original model (Imam and Cleland, 2019) while enabling a more sophisticated development of learned classes within the high-dimensional projection field. Even in its present feedforward form, however, the *EPLff* algorithm exhibits (1) rapid, online learning of arbitrary sensory representations presented in arbitrary sequences, (2) generalization across concentrations, (3) robustness to substantial changes in the diversity and responsivity of sensor array input without requiring network reparameterization, and, by virtue of these properties, is capable of (4) effective adaptation to ongoing sensor drift via a rapid reset-and-retraining process termed reset learning. This capacity for fast reset learning represents a practical strategy for field-deployable devices, in which a training sample kit could be quickly employed in the field to retune and restore functionality to a device in which the sensors may have degraded. Importantly for such purposes, the *EPLff* algorithm was not, and need not be, crafted to the statistics of any particular data set, nor was the network pre-exposed to testing set data as has been done in some approaches (Zhang and Zhang, 2015; Yan et al., 2017).

Because field-deployable devices require a level of generic readiness for undetermined or underdetermined problems, and these *EPLff* properties favor such readiness, we have emphasized the portability of these algorithms to neuromorphic hardware platforms that may come to drive such devices. Interestingly, many of the features of the biological olfactory system that have inspired this design are appropriate for such devices. Spike timing and event-based algorithms are attractive candidates for compact, energy-efficient hardware implementation (Imam et al., 2012; Merolla et al., 2014; Qiao et al., 2015; Diehl et al., 2016; Esser et al., 2016; Davies et al., 2018). Spike timing metrics can compute similar transformations as analog and rate-based representations; indeed, it has been proposed that spike based computations could in principle exhibit all of the computational power of a universal Turing machine (Maass, 1996, 2015). STDP is a localized learning algorithm that is highly compatible with the colocalization of memory and compute principle of neuromorphic design, and its theoretical capacities have been thoroughly explored in diverse relevant contexts (Nessler et al., 2009; Linster and Cleland, 2010; Schmiedt et al., 2010; Bengio et al., 2015; O'Connor et al., 2018). Our biologically constrained approach to algorithm design also provides a unified and empirically verified framework to investigate the interactions of these various algorithms and information metrics, to better interpret and apply them to artificial network design.

Other groups have previously proposed networks for gas sensor data analysis inspired by biological olfactory systems. Models of olfactory bulb and piriform cortical activity have been applied to analyze chemosensor array data (Raman and Gutierrez-Osuna, 2005; Raman et al., 2006). Algorithms based on the insect olfactory system have been employed to learn and identify odor-like inputs (Diamond et al., 2016; Delahunt et al., 2018) as well as to identify handwritten digits—visual inputs incorporating additional low-dimensional structure (Huerta and Nowotny, 2009; Delahunt and Kutz, 2018; Diamond et al., 2019). More broadly, insect mushroom bodies in particular have been deeply studied in terms of both their pattern separation and associative learning capacities (Hige, 2018; Cayco-Gajic and Silver, 2019). These capacities potentiate one another in service to odor learning and the classification of learned odor-like signals, though they also have been applied to more complex tasks (Ardin et al., 2016; Peng and Chittka, 2017). In the present work, we sought to design artificial learning networks to replicate some of the most powerful capabilities of the biological olfactory system, in particular its capacity for rapid online learning and the fast and effective classification of learned odorants despite ongoing changes in sensor properties and the unpredictability of odor concentrations. Future work will extend this framework to incorporate the feedback dynamics of the biological system, increase the dimensionality of sensor arrays, and develop more sophisticated biomimetic classifiers.

## Author Contributions

TC originally conceived the algorithm, which was vetted and modified for present purposes by AB and TC. AB designed, programmed, and performed the simulations. AB and TC designed the figures and wrote the paper.

### Conflict of Interest Statement

Both authors are listed as inventors on a Cornell University provisional patent (8631-01-US) covering other aspects of this algorithm.
